# Improved Inflammatory Balance of Human Skeletal Muscle during Exercise after Supplementations of the Ginseng-Based Steroid Rg1

**DOI:** 10.1371/journal.pone.0116387

**Published:** 2015-01-24

**Authors:** Chien-Wen Hou, Shin-Da Lee, Chung-Lan Kao, I-Shiung Cheng, Yu-Nan Lin, Sheng-Ju Chuang, Chung-Yu Chen, John L. Ivy, Chih-Yang Huang, Chia-Hua Kuo

**Affiliations:** 1 Laboratory of Exercise Biochemistry, Department of Sports Sciences, University of Taipei, Taipei, Taiwan; 2 Department of Physical Therapy, Graduate Institute of Rehabilitation Science, China Medical University, Taichung, Taiwan; 3 Department of Healthcare Administration, Asia University, Taichung, Taiwan; 4 Department of Physical Medicine and Rehabilitation, Taipei Veterans General Hospital, Taipei, Taiwan; 5 School of Medicine, National Yang-Ming University, Taipei, Taiwan; 6 Department of Physical Education, National Taichung University of Education, Taichung, Taiwan; 7 Department of Kinesiology and Health, University of Texas at Austin, Austin, Texas, United States of America; 8 Graduate Institute of Basic Medical Science, China Medical University, Taichung, Taiwan; 9 Department of Health and Nutrition Biotechnology, Asia University, Taichung, Taiwan; University of the Balearic Islands, SPAIN

## Abstract

The purpose of the study was to determine the effect of ginseng-based steroid Rg1 on TNF-alpha and IL-10 gene expression in human skeletal muscle against exercise challenge, as well as on its ergogenic outcomes. Randomized double-blind placebo-controlled crossover trials were performed, separated by a 4-week washout. Healthy young men were randomized into two groups and received capsule containing either 5 mg of Rg1 or Placebo one night and one hour before exercise. Muscle biopsies were conducted at baseline, immediately and 3 h after a standardized 60-min cycle ergometer exercise. While treatment differences in glycogen depletion rate of biopsied quadriceps muscle during exercise did not reach statistical significance, Rg1 supplementations enhanced post-exercise glycogen replenishment and increased citrate synthase activity in the skeletal muscle 3 h after exercise, concurrent with improved meal tolerance during recovery (P<0.05). Rg1 suppressed the exercise-induced increases in thiobarbituric acids reactive substance (TBARS) and reversed the increased TNF-alpha and decreased IL-10 mRNA of quadriceps muscle against the exercise challenge. PGC-1 alpha and GLUT4 mRNAs of exercised muscle were not affected by Rg1. Maximal aerobic capacity (VO_2max_) was not changed by Rg1. However, cycling time to exhaustion at 80% VO_2max_ increased significantly by ~20% (P<0.05). Conclusion: Our result suggests that Rg1 is an ergogenic component of ginseng, which can minimize unwanted lipid peroxidation of exercised human skeletal muscle, and attenuate pro-inflammatory shift under exercise challenge.

## Introduction

As a popular worldwide herbal supplement, ginseng has been alleged to have performance-enhancing properties for thousands of years. However, previous scientific studies present mixed results [1–4]. One major limitation in ginseng research is its inconsistent ginsenoside profile due to differences in variant type, harvesting season, and cultivated soil [5, 6]. To circumvent such shortcomings, bioactive components of ginseng, which can modulate metabolism and physical performance, need to be identified and studied independently in order to standardize ginseng and make it a reliable ergogenic or health-promoting nutraceutical.

Ginsenosides as a class of glycosylated steroids found in many types of ginseng have been the major target of ginseng research in recent years. However, the existing ginsenoside reports on physiological and metabolic functions are mostly limited in animal studies or *in vitro* cultured human cell models. To date, empirical evidence demonstrating ginsenosides can improve human physical performance and metabolism is unavailable. It has been documented in one animal study that 4 days of ginseng saponin supplementation (10 and 20 mg/kg/day) can significantly increase endurance performance of rats exercising at approximately 70% of maximal oxygen consumption (VO_2max_). Ginseng saponin devoid of Rg1 and Rb1 failed to demonstrate this positive effect, suggesting that these major steroidal compounds of ginseng may be responsible for the performance-enhancing attribute [7].

Exercise challenge inevitably generates unwanted molecular modification on cellular lipid components as a result of increasing metabolic stress. Increasing lipid peroxidation could result in compromised mitochondria function and carbohydrate metabolism [8, 9]. In a previous study involving race horses, a close association between pre-competition TBARS (biomarker of lipid peroxidation) level and endurance performance was reported, suggesting that accumulation of oxidized lipid components can undermine physical performance [10]. Therefore, minimizing such molecular attrition of lipid components in skeletal muscle during intensive exercise is essential for maintaining uncompromised metabolic function, physical performance, and post-exercise recovery/adaptation. Although the free radical scavenging effect of ginseng has been documented in several *in vitro* studies [11, 12], no existing literature has reported whether ginsenosides from ginseng contribute to the effect in exercised human skeletal muscle.

Oxidation-specific epitopes of lipids are danger-associated molecular patterns (DAMP) of cells that trigger inflammation [13]. Once inflammation is triggered, pro-inflammatory cytokines TNFɑ and IL-6 are released from challenged tissue to orchestrate the local inflammation events aiming to clear damaged cells and reconstruct the tissues [14]. In contrast, the anti-inflammatory cytokines IL-10 inhibits the inflammation process when the tissue is sufficiently restored [15–17]. Thus, inflammatory balance can reflect the health status or robustness of skeletal muscle against an acute bout of physical challenge [17]. In this study, inflammatory responses were measured to evaluate the influence of pre-workout ginsenoside Rg1 supplementations on the coping capability of human skeletal muscle against an exercise challenge.

Here, we hypothesized that Rg1 can improve endurance performance at high intensity exercise and modulate inflammatory balance in exercised skeletal muscle. Randomized, double-blind, placebo-controlled, crossover trials were conducted to evaluate TNF-alpha and IL-10 mRNAs in the exercised human skeletal muscle and the ergogenic outcomes, including aerobic fitness and endurance performance at 80% VO_2max_, after pre-workout Rg1 supplementations. Training adaptation markers of skeletal muscle, including citrate synthase activity and glycogen storage rate from biopsied *vastus lateralis* were also determined immediately and 3 h after a standardized 60-min exercise.

## Methods

### Ginsenosides Rg1

**Figure 1:**
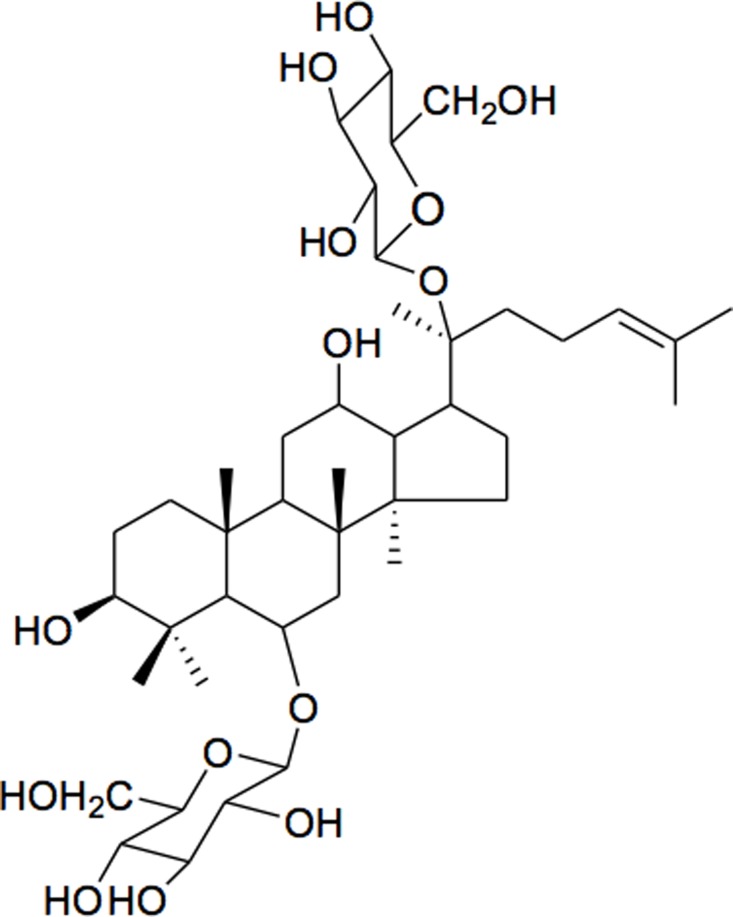
Chemical structures of Rg1.

Rg1 (NuLiv Science, Inc., Walnut, California, USA) is a major ginsenoside compound of *Panax Notoginseng*, which has a steroid core structure and covalently bonded with sugars (Fig. 1), also existing in most species of ginseng.

### Ethics statement

Human trials, which include inform consent procedure, were approved by Institutional Review Board of The University of Taipei before the study began. This study was conducted in accordance with the guidelines in the Declaration of Helsinki. All subjects were well informed of the potential risks. Participants provide their written informed consent to participate in this study. Exclusion criteria were history of musculoskeletal injury or receiving medication in the previous 1 month.

### Experimental designs

Three trials were conducted to evaluate the ergogenic properties of Rg1. One night prior to the exercise challenge (~12 h), all subjects were instructed to take a standard diet (plus can of Ensure) along with their first dose of either Rg1 or placebo capsule provided by a research assistant in the laboratory at University of Taipei. For each trial, subjects consumed a bottle of mineral water (Taiwan Yes Deep Ocean Water Co., Ltd., Hualien, Taiwan) on the day of the experiment, and 1-h prior to the exercise challenge when subjects received their 2^nd^ dose of Rg1 or Placebo.

Research committee recommended that the group receives 50–100% of the lowest dose of Rg1 according to preliminary animal results, which generates the most favorable glucose tolerance. Each Rg1 capsule contains 5 mg of Rg1 with flour powder, whereas placebo capsule contains same amount of flour. Effects of Rg1 were always evaluated in a randomized, double-blind, placebo-controlled, crossover fashion with a 4-week washout period to remove possible residual effects of oral ginsenoside intake. Participants were randomly assigned to one of two parallel groups, initially in 1:1 ratio, to receive either Rg1 or Placebo under supervision of a staff to ensure subject compliance. The Rg1 and Placebo were in capsule form with identical in appearance. They were placed in a container and consecutively numbered for each participant according to the randomization schedule. Each participant was assigned an order number and received the capsules in the corresponding bag. A computer-generated list of random numbers was used for allocation of the participants. Project manager enrolled participants. A research staff assigned participants to interventions. Except for the interventionists, investigators and staff were kept blind to supplement assignment of the participants. The trial maintained separation between staff that take outcome measurements and staff that deliver the intervention. Staffs and participants were kept masked to outcome measurements and trial results.

Trial 1 (n = 14, untrained healthy men, age 21.1±1.3 years, weight 72.6±7.8 kg, height 176.6±1.0 cm, VO_2max_ 3.1±0.2 L/min) was designed to measure glycogen recovery of skeletal muscle and meal tolerance after 1 hour of exercise at 70% VO_2max_. Trial 2 (n = 12, untrained healthy men, aged 22.2 ± 0.6 years, weight 72.1±2.3 kg, height 175.2±0.1cm, VO_2max_ 3.6±0.3 L/min) was conducted to determine aerobic fitness (maximal oxygen consumption) and endurance performance (cycling time to exhaustion at 80% VO_2max_) after Rg1 supplementations. Trial 3 (the same participants as Trial 2) was conducted to measure oxidative damage (TBARS), inflammatory markers, and citrate synthase activity of skeletal muscle during recovery following 1 hour of exercise at 70% VO_2max_.

### Muscle biopsy

For Trail 1 and 3, all subjects performed an acute bout of 60-min of cycling at 70% VO_2max_ and were observed during their first 3 h of recovery. *Vastus lateralis* samples were collected using a percutaneous biopsy technique under local anesthesia. An 18-G Temno disposable cutting needle (Cardinal Health, McGaw Park, Illinois, USA) was inserted into the vastus lateralis positioned at 1.0–1.5 cm depth, ~20 centimeter proximal to knee cap. Muscle samples were taken at baseline (designated as Pre: 2 weeks before the exercise challenge), immediately after and 3 h after exercise. They were frozen by liquid nitrogen immediately after isolation. Muscle samples were homogenized (1:20, red muscle and 1:15, white muscle) in 20 mM ice-cold N-2-hydroxyethylpiperazin-N’-2-ethanesulfonic acid, 1 mM EDTA, and 250 mM sucrose (HES) buffer (pH 7.4) with a Polytron (Brinkman Instruments, Westbury, NY) for measurements of citrate synthase activity and TBARS levels.

### Maximal oxygen consumption (VO_2max_)

Vo_2max_ was measured one week before exercise challenges on a Monark cycle ergometer (Monark Ltd, Varberg, Sweden), using a continuous incremental cycle ergometer protocol. This protocol consisted of a 5-min warm up and incremental increases in workload of 60 W every 3 min until exhaustion. VO_2max_ was verified by a respiratory exchange ratio (RER) of greater than 1.1 and a plateauing of VO_2_ with increasing workload. O_2_ and CO_2_ concentrations of inspired and expired gases were measured with a MetaMax I system (Leipzig, Germany).

### Endurance performance

We determined the influence of pre-workout Rg1 supplementations on high intensity endurance performance at 80% VO_2max_. Participants were asked to pedal on the same Monark cycle ergometer used for their VO_2max_ measurement until exhaustion. During the exercise test, subjects were asked to keep the rotational speed at 60 rpm to their best effort. After warm-up for 5 min at a work rate (watt) equivalent to 60% VO_2max_, the work rate was increased to 80% VO_2max_. Fatigue was defined as the point at which subjects could no longer maintain at the designated intensity on the cycle ergometer.

### Meal tolerance test

Meal tolerance was evaluated after the exercise challenge along with measurement of muscle glycogen storage, according to the method published previously [18]. A standardized meal consisting of 70% carbohydrate (glycemic index: 77; total energy intake: 689 Kcal) was received by all participants (2 g carbohydrate per kilogram of body weight). This meal contained corn flakes (Kellogg’s Ltd, Manchester, UK), skimmed milk, bread, jam and water (carbohydrate, 140.1 g; protein, 19.7 g; fat, 5.5 g) prepared by a registered dietitian. Biopsied muscle samples were obtained immediately and 3 h after exercise for determination of post-exercise recovery in glycogen, oxidative damage (TBARS), citrate synthase activity, and inflammatory markers. Blood glucose and insulin were also determined during the 3-h recovery.

### Blood analysis

For the meal tolerance test during trial, a 20-G polyethylene catheter (Jelco, Tampa, FL, USA) was placed in an antecubital vein for blood sampling. Blood samples were then taken before and after meal consumption. During recovery, blood samples were also collected every 30 min for 180 min. The catheter was kept paten by flushing with a small amount of saline solution containing heparin (10 IU/ml) following each sample collection. Blood samples were collected into fluoride heparin and plasma tubes. Plasma was obtained after centrifuging at 4°C for 10 min at 3000 rpm and was stored at -80°C before analysis. Blood glucose was determined by an automated glucose analyzer (YSI Life Sciences, Yellow Springs, OH, USA). Plasma insulin levels were determined by using the radioimmunoassay method with a commercial kit (Baylor Diagnostics, Tarrytown, NY, USA) according to the manufacturer’s instructions.

### Glycogen assay

A small amount of skeletal muscle from the *vastus lateralis* muscle was dissolved in 1 N KOH at 75°C for 30 min. Dissolved samples were neutralized by glacial acetic acid. They were incubated overnight in acetate buffer (0.3 M sodium acetate, pH to 4.8) containing amyloglucosidase (Boehringer Mannheim, Indianapolis, IN) for degradation of glycogen into glucose. The reaction mixture was neutralized with 1 N NaOH. Samples were then analyzed by measuring glucosyl units by the Trinder reaction (Sigma, St. Louis, MO).

### Quantitative polymerase chain reaction (qPCR)

Total RNA from biopsied muscle tissue was extracted using RNeasy Fibrous Tissue Mini Kit (Qiagen, Valencia, CA, USA) according to the manufacturer′s instructions. The extracted RNA was dissolved in RNase free water. Reverse transcription reactions were performed on extracted RNA using an iScript cDNA synthesis kit (Bio-Rad Labatories, Inc., Herciles, CA, USA) in a reaction volume of 40 μl. Primers and TaqMan probes were designed using BioRad Beacon Designer (BioRad Labatories, Herciles, CA, USA). All primers and TaqMan probes were supplied from the same vender (Sigma Proligo, Singapore). We quantified gene expression against 18S rRNA as a reference gene, using a critical threshold (CT) method using the MyiQ real-time PCR detection system (BioRad Labatories, Herciles, CA, USA). A CT value reflects the cycle number at which the DNA amplification is first detected. PCR amplification was carried out in 25 μl reactions of iQ supermix, 400 nM forward primer, 400 nM reverse primer, and 300 nM TaqMan probe. Each reaction was made up to volume with DNase-free water. Fold changes against control were calculated using the 2−ΔΔCT method. For amplification reaction, the oligonucleotide primer and probe sequences of 18S rRNA, TNF-α, IL-6, IL-10, GLUT4, and PGC-1α genes were synthesized by MDBio (Taipei, Taiwan). The primer and probe sequences for the 18S rRNA gene (NM_001042) were 5′- CACAGTCTTCACCTTGGTCTCG -3′ (forward), 5′- CCAGGGACCTCTCTCTAA -3′ (reverse), and 5′- GCTACAACATGGGCTACA -3′ (Probe). The primer and probe sequences for the TNFα gene (NM_000594) were 5′- CCAGGGACCTCTCTCTAA -3′ (forward), 5′- GCTACAACATGGGCTACA -3′ (reverse), and 5′- CCAGGCAGTCAGATCATCTTCTCG -3′ (Probe). The primer and probe sequences for the IL-6 gene (NM_000600) were 5′- TTCGGCAAATGTAGCATG -3′ (forward), 5′- CAGTGGACAGGTTTCTGA -3′ (reverse), and 5′- CCATTAACAACAACAATCTGAGGTGC -3′ (Probe). The primer and probe sequences for the IL-10 gene (NM_000572) were 5′- CTTCCCTGTGAAAACAAG -3′ (forward), 5′- AGACCTCTAATTTATGTCCTA -3′ (reverse), and 5′- AGTCGCCACCCTGATGTCTC -3′ (Probe). The primer and probe sequences for the GLUT4 gene (NM_001042) were 5′- CACAGTCTTCACCTTGGTCTCG -3′ (forward), 5′- GTAGCTCATGGCTGGAACTCG -3′ (reverse), and 5′- CCAGCAGGAGCAGAGCCACAGTCA -3′ (Probe). The primer and probe sequences for the PGC-1α gene (NM_013261) were 5′- CGAGGAATATCAGCACGAGAGG -3′ (forward), 5′- CATAAATCACACGGCGCTCTTC -3′ (reverse), and 5′- TGCCTTCTGCCTCTGCCTCTCCCT -3′ (Probe).

### Oxidative damage (Thiobarbituric acid reactive substances)

Homogenized muscle samples were diluted for TBARS measurement as a byproduct of lipid oxidation. TBARS was determined by ELISA using a TBARS assay kit (Cayman Chemical Company, Ann Arbor, MI, USA).

### Citrate synthase activity

Muscle homogenate was used for determination of citrate synthase activity. After further 1: 10 dilution with 0.1 M Tris-HCl and 0.4% Triton X-100 buffer, citrate synthase activity was measured spectrophotometrically on the homogenates according to Srere [19].

### Statistical analysis

Shapiro-Wilk test was used to test whether the data were normally distributed. Homogeneity of variances was confirmed by Levene’s test. Comparisons between paired data were analyzed using the non-parametric Wilcoxon′s signed rank test. To analyze changes over time, a Friedman test was used and when a significant F-ratio was found, Wilcoxon’s signed rank test was used for post hoc analysis. A level of significance was set at P < 0.05, and all values are expressed as means ± standard errors.

## Results

Three trials were performed to determine the ergogenic properties of Rg1. No participant experienced any adverse events due to the Rg1 treatment.

**Figure 2:**
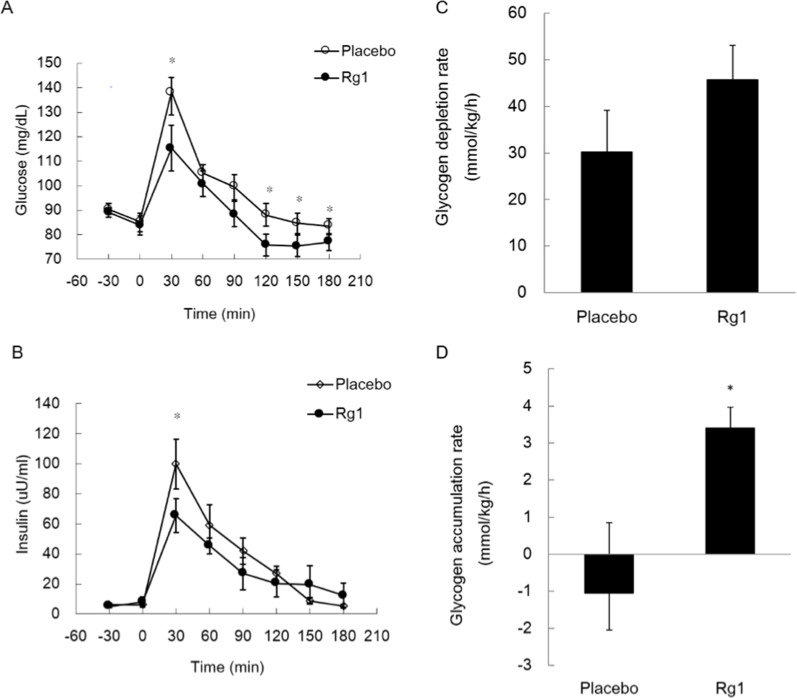
Oral Rg1 supplementations accelerated glycogen recovery in human skeletal muscle after an acute bout of 1 h exercise (70% VO_2max_). Rg1 was orally delivered 1 night and 1 h before exercise. Glucose (A) and insulin (B) levels in blood after a post-exercise meal. Glycogen depletion rate during 1 h exercise (C) and glycogen accumulation rate (D) during 3 h recovery in *vastus lateralis* muscle. * Significantly different from Placebo trial, P< 0.05.

Trial 1 evaluated the effect of pre-workout Rg1 supplementations on glycogen recovery in skeletal muscle after a 60-min exercise at 70% VO_2max_ (Fig. 2). A meal tolerance test (80% high carbohydrate) was performed during a 3-h recovery period. Significantly lowered glucose (Fig. 2A) and insulin (Fig. 2B) levels following the post-exercise meal were observed after Rg1 administration compared to Placebo. Glycogen accumulation rate was assessed by measuring glycogen concentration on two biopsies from the *vastas lateralis* at 0 and 3 h after the exercise. The rate of glycogen depletion was not significantly different between the Rg1 and Placebo trials (Fig. 2C). However, a significantly accelerated rate of glycogen accumulation in exercised skeletal muscle was observed after Rg1 administration (Fig. 2D).

Trial 2 was conducted to evaluate endurance performance at high intensity. There was no significant difference in VO_2max_ between the Rg1 and Placebo trials. However, Rg1 significantly increased exercise time to exhaustion (Rg1: 38.3±6.7 min versus Placebo: 31.8±5.0 min) during a cycle ergometer test at 80% VO_2max_ (Table 1).

**Table 1:** Ergogenic property of Rg1. Cycling time to exhaustion significantly increased by oral Rg1 supplementations without changes in maximal aerobic capacity in untrained healthy men.

**Performance measures**	**Placebo**	**Rg1**	***P***
Cycling time to exhaustion (min)	31.8±5.0	38.3±6.7	0.04
Total work (kJ)	254±41	306±55	0.04
VO_2max_ (ml/kg/min)	41.6±2.4	40.3±1.9	NS

**Figure 3:**
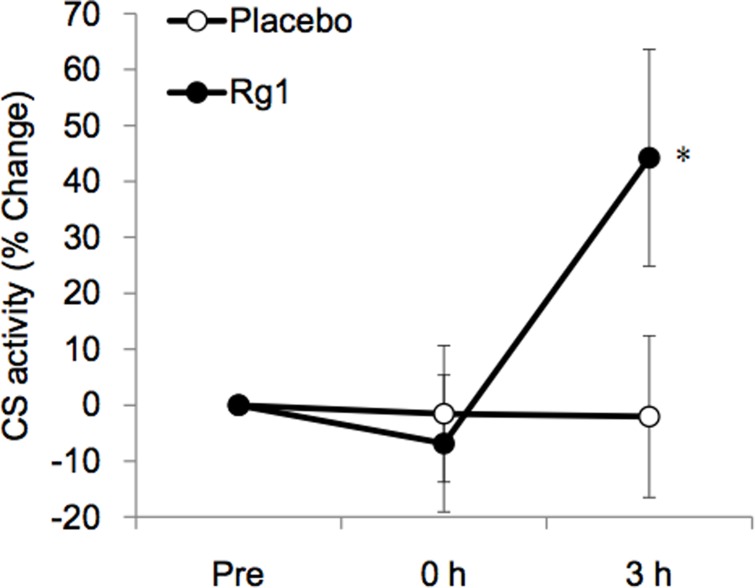
Mitochondria enzyme adaptation in exercised skeletal muscle is facilitated by oral Rg1 supplementations. Citrate synthase (CS) activity of *vastus lateralis* muscle after exercise. * Significantly different from Placebo trial, P< 0.05.

**Figure 4:**
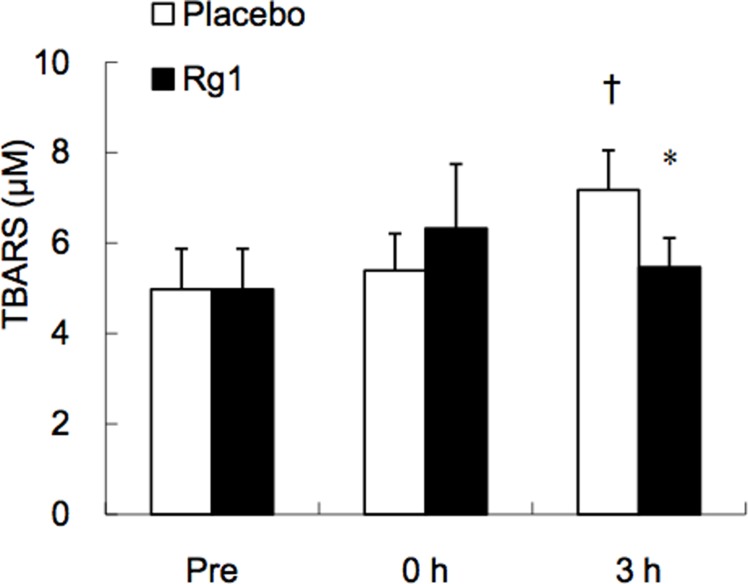
Elevated lipid peroxidation in exercised human skeletal muscle is suppressed by oral Rg1 supplementations. Thiobarbituric acid reactive substances (TBARS) in *vastus lateralis* muscle after exercise. * Significantly different from Placebo trial, P< 0.05; † Significantly different from Pre, P< 0.05.

**Figure 5:**
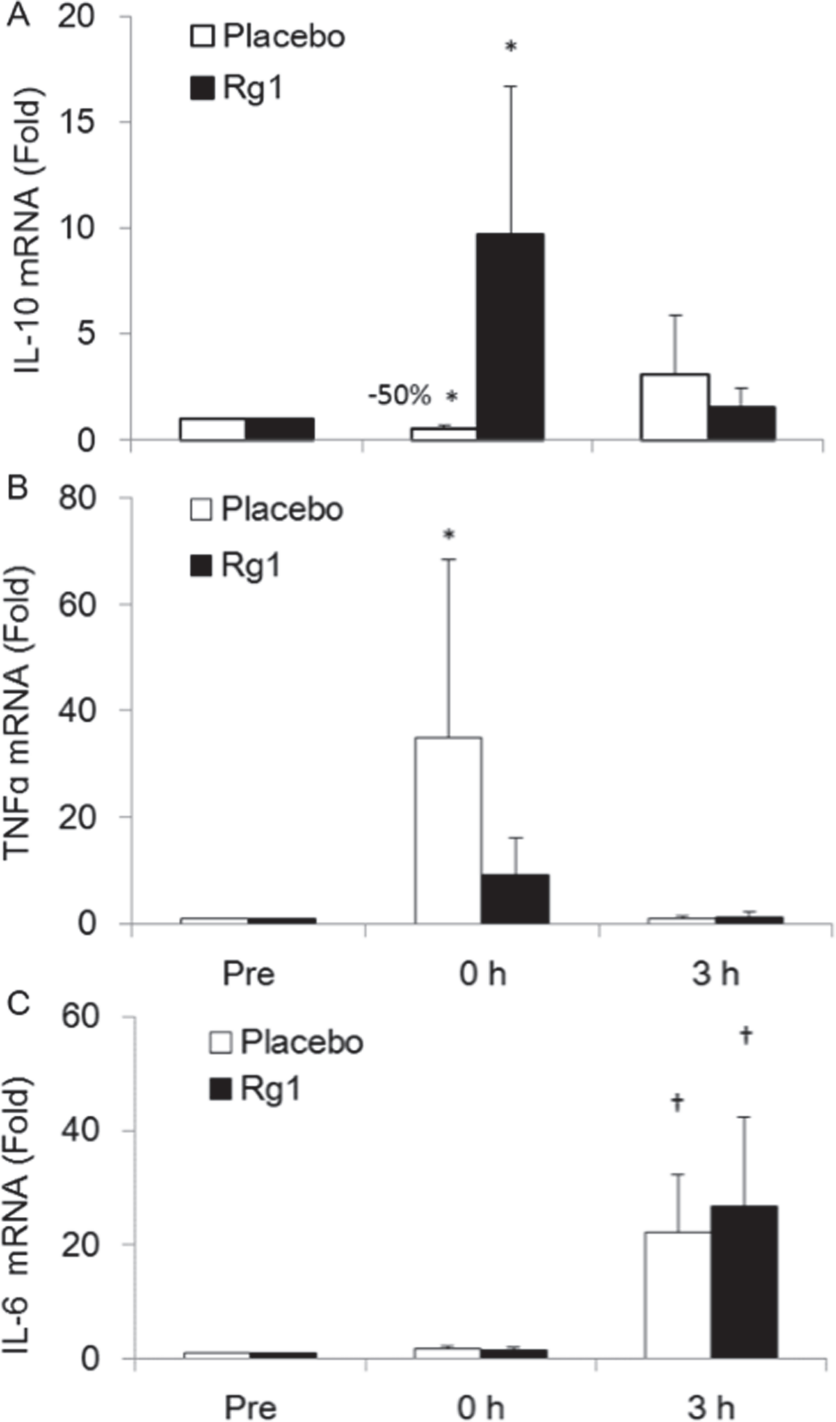
Oral Rg1 supplementations reverses pro-inflammatory shift in balance of exercised human skeletal muscle. Quantitative PCR data of mRNAs for TNF-α (A), IL-10 (B), and IL-6 (C) in *vastus lateralis* muscle.

Trial 3 evaluated the mitochondria enzyme adaptation (Fig. 3), lipid peroxidation (Fig. 4), and inflammatory potentiation (Fig. 5) of exercised skeletal muscle during recovery. Results from the biopsied muscle samples reveal that Rg1 supplementations induced a significant increase in citrate synthase (CS) activity within only 3 h following exercise at 70% VO_2max_, whereas this increase was not detected for the Placebo trial. The lipid peroxidation marker TBARS in exercised human muscle increased significantly during the Placebo trial. However, this increase was eliminated completely during the Rg1 trial (Fig. 4). Exercise significantly lowered IL-10 mRNA levels by ~50% (Fig. 5A) with increased TNF-α mRNA (Fig. 5B). This pro-inflammatory shift in balance induced by exercise was reversed by pre-workout Rg1 supplementations. A delayed increase in IL-6 mRNA was found 3 h after exercise (Fig. 5C, P< 0.05) without an observable difference between Rg1 and Placebo trials.

**Figure 6:**
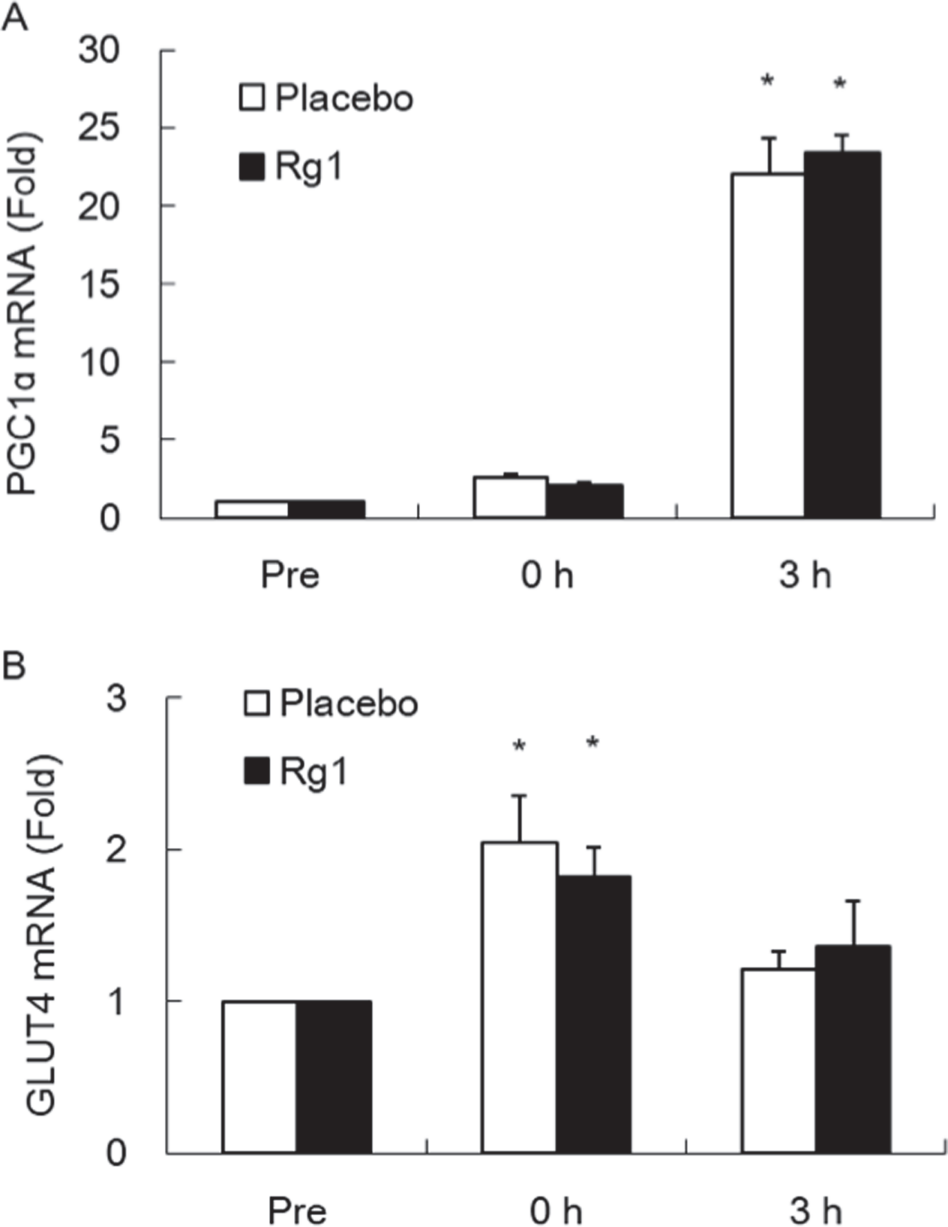
PGC-1α and GLUT4 expressions after exercise are not influenced by oral Rg1 supplementations. Quantitative PCR data of mRNAs for PGC-1α (A) and GLUT4 (B) in *vastus lateralis* muscle. * Significantly different from Placebo trial, P< 0.05.

Exercise significantly increased PGC-1α and GLUT4 mRNAs (P<0.05). However, no significant differences in PGC-1α (Fig. 6A) or GLUT4 (Fig. 6B) mRNAs were found between Rg1 and Placebo trials.

## Discussion

The major finding of the study is that pre-workout Rg1 supplementations significantly increased endurance performance at high exercise intensity. These untrained participants were able to undertake approximately 20% additional work on a cycle ergometer compared with Placebo trial. Muscle contraction unavoidably increases oxidative attrition on cellular components, as shown by increased TBARS as a byproduct of lipid peroxidation, which can result in a transient weakening of cellular function. Thus, the improvement in endurance performance by Rg1 could be associated, in part, with better preservation of native lipid structure in skeletal muscle during exercise.

Optimization of physical performance demands uncompromised metabolic function in the maintenance of ATP regeneration, substrate replenishment, and tissue repair during and after exercise. Here we observed relative to a placebo control a suppressed accumulation of lipid peroxidation in exercised skeletal muscle after Rg1 administration, coincident with enhanced meal tolerance, glycogen accumulation, and mitochondria enzyme adaptation during recovery. Increasing lipid peroxidation is well-known to cause a fundamental impact on metabolic function, such as decreased insulin sensitivity of skeletal muscle [9] and deterioration in mitochondria function [8]. Thus, results of the study suggest that improved endurance performance and accelerated functional recovery of skeletal muscle are associated with the buffering action of Rg1 against oxidative stress induced by exercise.

Accumulated oxidative damage is a form of DAMP recognized by receptors of innate immunity to initiate inflammation [13]. Along with an increase in lipid peroxidation following exercise, we found significantly increased TNFɑ mRNA and decreased IL-10 mRNA in human skeletal muscle, indicating an inflammatory potentiation. This potentiation is eliminated after pre-workout Rg1 supplementations. The elimination of this pro-inflammatory shift by Rg1 administration appeared to be associated with elimination of TBARS after exercise. Inflammation is a mechanism of multicellular organisms to initiate the local healing process in response to traumatic stimuli by removing unhealthy cells and triggering repopulation of the tissue. IL-10 has been considered an anti-inflammatory cytokine that functions to inhibit early stage of inflammation process when the tissue is returning healthy. The result of the study demonstrates that Rg1 can modulate inflammation status of human skeletal muscle after an acute bout of exercise. Thus, this observed response after Rg1 administration might be associated with a decreased production of oxidation-specific epitopes in exercised skeletal muscle.

An increase in citrate synthase activity in skeletal muscle is widely considered an indication of increased mitochondria density following exercise training. This adaptive response takes place slowly and is not generally observed within 3 h of post-exercise recovery. Therefore, Rg1 administration may stabilize the infrastructure of exercised muscle, reduce the need for tissue repair, and pave the way for a faster functional recovery and adaptation.

It is unclear how Rg1 protects lipid components of skeletal muscle against exercise-induced oxidative stress. We speculate that the antioxidant effect of Rg1 may be a result of plasma membrane stabilization of skeletal muscle against exercise. After a traumatic challenge, activated macrophage during inflammation can further increase local free radicals. Decreased membrane fluidity is associated with increased oxidative stress [21]. Plasma membrane is an important site where cell signaling is taking place. A previous *in vitro* study reported an increased membrane fluidity after Rg1 treatment [20]. We propose that incorporation of the bulky steroid moiety of Rg1 into cellular membrane lipid may enhance molecular complexity and mechanical stability of the cell and mitochondrial membranes against temperature shifts and ionic leakage across membranes during a changing rate of oxidation [22, 23]. Temperature shifts typically occurs during and after exercise. Thus, molecular stabilization of membrane lipid structures after Rg1 supplementations may increase the robustness of skeletal muscle to withstand the challenged created by temperature shift during exercise. This buffering action may preserve the health of skeletal muscle, which is essential for adaptive response in both mitochondria biogenesis and transmembrane insulin signaling system.

It is surprising that no glycogen accumulation occurs during the 3 h recovery in Placebo trial. The depth of needle biopsy has been controlled to superficial area. In animals, muscle fiber composition varied markedly between deep and superficial muscle samples [24], which may affects glycogen turnover. Superficial muscle fiber containing more type 2 fiber, which tends to be less insulin sensitive in glycogen storage and thus may explain why glycogen of exercised muscle has not been recovered in 3 h.

In conclusion, our study discovers that the ginseng-based steroid Rg1 can offer ergogenic benefits for humans. The accelerated post-exercise recovery and mitochondria enzyme adaptation after Rg1 pre-treatment appears to be mediated by its capability to buffer exercise-induced oxidative stress in human skeletal muscle. This is evidenced by the observation that Rg1 suppressed an increase in TBARS and attenuated a pro-inflammatory shift caused by prolonged exercise challenge.
